# Adsorption of water on pristine graphene: a van der Waals density functional study with the vdW-C09 approach

**DOI:** 10.1007/s00894-026-06663-1

**Published:** 2026-03-03

**Authors:** Aline Oliveira Santos, Bruno H. S. Mendonça, Elizane E. de Moraes

**Affiliations:** 1 Instituto de Física, Universidade Federal da Bahia, Campus Universitário de Ondina, Salvador, 40210-340 Bahia Brazil; 2 Departamento de Física, ICEX, Universidade Federal de Minas Gerais, Av. Pres. Antônio Carlos, 6627, Belo Horizonte, 31270-901 Minas Gerais Brazil

**Keywords:** Graphene-based membranes, Water–surface interaction, Nanofluidics, Adsorption modeling, Environmental nanotechnology

## Abstract

**Context:**

Understanding how water interacts with graphene at the molecular level is essential for advancing nanomaterial applications in filtration, catalysis, and environmental technologies. This study establishes a quantitative baseline for assessing how structural defects, dopants, or surface functionalization may enhance water adsorption, providing insights for the rational design of graphene-based materials in water purification, sensing, and nanofluidic applications.

**Methods:**

In this work, we employed density functional theory (DFT) with the vdW-C09 functional to investigate the adsorption of a single water molecule on pristine graphene, accurately accounting for long-range dispersion forces. Three high-symmetry adsorption sites—the center of the hexagonal ring, the C–C bond, and the top site—were explored in combination with three molecular orientations: Down, H-bond, and Up configurations. The calculated adsorption energies range from −93 to −145 meV, indicating that the interaction is dominated by weak van der Waals forces characteristic of physisorption. The most stable configuration corresponds to the Down orientation above the center of the hexagonal ring, with an adsorption energy of −145 meV and an equilibrium distance of 3.27 Å defined as the vertical separation between the oxygen atom of the water molecule and the graphene plane. These results are in close agreement with previous theoretical studies and confirm the non-reactive and hydrophobic nature of pristine graphene.

## Introduction

Due to its remarkable electronic, mechanical, and surface area properties, graphene has become the foundation of modern nanotechnology [1–7]. In particular, its application in aqueous systems, such as desalination membranes, advanced nanofilters, and environmental catalysis devices, demonstrates revolutionary potential in the field of water treatment [8–13]. However, the performance and efficiency of these nanodevices are intrinsically dictated by the fundamental nature of the interface between water and graphene. The precise interaction of the water molecule with the inert, nonpolar graphene surface is a key factor in controlling wettability, nanoscale fluid transport, and the mechanism of contaminant adsorption [14–22].

Although important, accurately describing the interaction between water and graphene remains a theoretical challenge [23–25]. The predominant interactions are weak, non-covalent dispersion forces, commonly referred to as van der Waals forces. Traditional Density Functional Theory (DFT) methods often do not adequately represent these forces [26]. The lack of a thorough understanding of the preferred adsorption sites and molecular orientations of water on the graphene surface limits the rational optimization of carbonaceous materials. To provide a reliable microscopic image, it is crucial to utilize methodologies that accurately capture these long-range scattering effects [20, 25, 27–32].

Accurate determination of the interaction energy between water molecules and graphene, resulting from the subtle balance between water-surface adhesion forces and intermolecular hydrogen bonds, is essential for a realistic description of water behavior in graphene membranes [30, 33–39]. The water-graphene interaction is characteristically weak, yet it plays a decisive role in modulating the structural and dynamic properties of water confined within carbon nanostructures [40]. The main mechanism involved is the van der Waals (vdW) interaction, which governs the adsorption of water molecules on the graphene surface [41]. Additionally, electrostatic and polarization effects are relevant: the presence of adsorbed water molecules induces significant polarization in the carbon network, contributing to the overall attractive potential between water and graphene [42–44]. The weak nature of the adsorption is directly reflected in the electronic structure of graphene [33]. The presence of water molecules does not induce appreciable doping, and the system’s electronic bands remain practically unchanged compared to those of intrinsic graphene. Only a slight splitting of bands is observed, associated with symmetry breaking and the introduction of additional energy levels from the molecular orbitals of water [45–48].

The structural organization of water on surfaces or under confinement is governed by a delicate balance between adsorption interaction on the surface and intermolecular hydrogen bonds [40, 49–52]. Thus, the rigorous quantification of these interactions is a fundamental requirement for the accurate modeling of water adsorption on graphene, providing the necessary theoretical basis for understanding the ultrafast (or anomalous) flow of water in graphene membranes, which is the phenomenon that underpins the potential of these structures in advanced desalination and water purification processes [53–57].

Although the adsorption of water on graphene has been addressed in previous theoretical works, reported binding energies and equilibrium distances still vary depending on the treatment of dispersion interactions. In this study, we employ the vdW-C09 functional, which has been shown to provide an improved description of long-range van der Waals forces compared to standard GGA approaches, often reducing overbonding and distance inaccuracies. Our objective is not to introduce a new methodology, but to establish a consistent and well-characterized reference for water adsorption on pristine graphene, which can serve as a reliable baseline for future investigations of defected, doped, or functionalized graphene surfaces.

In this work, we therefore provide a systematic description of the adsorption of a single water molecule on a perfect graphene sheet using DFT with the vdW-C09 functional. We investigate three high-symmetry adsorption sites—the center of the hexagonal ring, the C–C bond, and the top site—and three distinct molecular orientations: down, H-bond, and up. By comparing adsorption energies and equilibrium distances, this study identifies the thermodynamically most favorable configurations and structural characteristics of water in contact with graphene. The results establish a validated reference dataset that contributes to a fundamental understanding of how nanoscale surface properties influence water behavior, providing a solid theoretical basis for the rational design of graphene-based materials for filtration, catalysis, and environmental nanotechnology.

## Computational details

Our calculations were based on the density functional theory (DFT) [58] as implemented in the SIESTA code version 4.1-b4 [59, 60]. We employed the well-known exchange–correlation functional of vDW-C09 [61]. Spin polarization was included in all calculations. We used the norm-conserving pseudopotentials in the Kleinman-Bylander factorized form [62, 63] with a double-zeta plus polarization (DZP) basis set for C, H, and O atoms. The real-space integration grid was defined by an energy cut-off of 350 Ry. Structural optimizations were carried out until the residual forces on all atoms were smaller than 0.05 eV/Å^*−*1^. Self-consistent field (SCF) calculations were considered converged when the total energy variation between successive iterations was below 10^*−*5^ eV, and the density-matrix variation was below 10^*−*5^.

Although the water–graphene system is formally a closed-shell system, spin polarization was included in all calculations to avoid artificial constraints and to allow for possible symmetry-breaking or subtle electronic rearrangements upon adsorption. In all cases, the final converged solutions corresponded to a non-magnetic ground state with zero net spin polarization.

A graphene supercell containing 144 carbon atoms was employed with periodic boundary conditions, and a vacuum region of approximately 15 Å was introduced along the out-of-plane direction to prevent spurious interactions between periodic images.

For the interaction energy calculations, we used the following equation through BSSE (basis set superposition error) corrected for all calculations with the counter-poise method:

1 $$E\:=\:E(A\:+\:B)\:-\:E(A\:+\:B_{ghost})\:-\:E(B\:+\:A_{ghost})$$

This correction is performed starting from the initial geometry of the AB system and calculating the total energy of system A, considering the whole set of base functions, where the set of base functions B is in the position corresponding to system B, without the explicit presence of the atoms. The same occurs in the calculation of system B. The system with negative binding energies implies an attractive interaction.

## Results and discussion

**Fig. 1:**
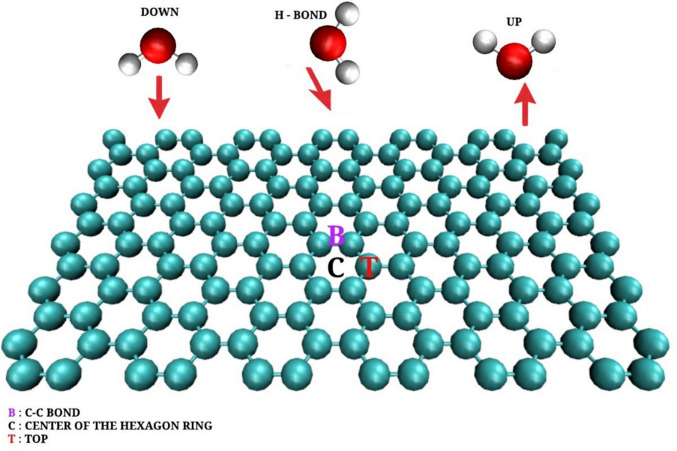
Optimized geometries of a single water molecule adsorbed on pristine graphene obtained from DFT calculations using the VDW-C09 functional. Three adsorption sites were considered: (a) center of the hexagonal ring, (b) C–C bond, and (c) top of a carbon atom. Oxygen atoms are shown in red, hydrogen in white, and carbon in blue

The interaction between a single water molecule and pristine graphene was investigated using DFT calculations within the VDW-C09 functional, which properly accounts for long-range dispersion forces. Three adsorption sites were considered: the center of the hexagonal ring, the C–C bond, and the top site, along with three molecular orientations: Down, H-bond, and Up, as seen in Fig. 1. The corresponding adsorption energies (*E*_ads_) and equilibrium distances between the oxygen atom of the water molecule and the graphene surface are summarized in Table 1. The equilibrium distance is defined as the vertical distance from the oxygen atom of a water molecule to the graphene plane, rather than the shortest O–C interatomic distance.

**Table 1:** Adsorption energies (*E*_ads_) and equilibrium distances (*d*) of a single water molecule on pristine graphene for different adsorption sites and molecular orientations, obtained from DFT calculations with the VDW-C09 functional

Orientation	Center of hexagonal ring		C–C bond		Top	
	*E*_ads_ (meV)	*d* (Å)	*E*_ads_ (meV)	*d* (Å)	*E*_ads_ (meV)	*d* (Å)
Down	−145	3.27	−141	2.80	−137	2.90
H-bond	−128	2.90	−133	2.60	−134	2.70
Up	−98	3.07	−94	3.10	−93	3.30

Our calculations reveal that the interaction between water and pristine graphene is weak, with adsorption energies ranging from −93 to −145 meV. Such values are characteristic of physisorption, confirming that water molecules adhere to graphene mainly through van der Waals dispersion forces. This finding aligns with previous reports and supports the hydrophobic character typically observed for pristine graphene surfaces. Among the three orientations considered, the Down configuration—where the hydrogen atoms of the water molecule point toward the graphene surface—was found to be the most stable, particularly when the molecule is placed over the center of the hexagonal ring (–145 meV). This preference can be understood in terms of the molecular dipole of water interacting with the polarizable *π*-electron cloud of graphene.

In the Down orientation, the hydrogen atoms, corresponding to the positive end of the dipole, are positioned closer to the surface, enhancing dipole–induced dipole and polarization contributions in addition to dispersion forces.

The H-bond configuration exhibits intermediate stability, with adsorption energies around −130 meV and slightly shorter equilibrium distances (down to 2.6 Å), consistent with weak local polarization effects but not indicative of chemical bonding. In contrast, the Up configuration, in which the oxygen atom faces the surface, is the least stable (−93 to −98 meV) and corresponds to the largest separations between the molecule and the graphene plane, reflecting a less favorable electrostatic alignment of the molecular dipole with the surface.

The equilibrium distances obtained (2.6–3.3 Å), together with the weak adsorption energies, are characteristic of physisorption dominated by van der Waals interactions. Moreover, the small energy differences among adsorption sites indicate that pristine graphene provides a nearly homogeneous, nonpolar surface with no strongly preferred binding positions. In this weak-binding regime, the interaction is nonreactive, with any electronic rearrangement limited to minor polarization effects rather than significant charge transfer or covalent bonding.

The adsorption energies obtained in this work are in excellent agreement with previous DFT studies that also describe water–graphene interactions as weakly bound systems governed by van der Waals forces [30, 33, 35, 39, 64]. Some works [35, 39] reported adsorption energies ranging from −100 to −150 meV and equilibrium distances around 3.0Åusing vdW-corrected functionals, consistent with our results. Similarly, works [30, 33, 64] found comparable values using PBE-D2 and optB88-vdW approaches, further confirming that the Down configuration—where the hydrogen atoms of the water molecule face the graphene surface—is the most stable orientation. In addition, experimental observations also support this weak physisorption behavior. Contact angle measurements and surface wetting studies on pristine graphene consistently indicate hydrophobic behavior, with contact angles typically above 85, implying only minor adhesion of water to the surface [65–67]. These findings rein-force that the water–graphene interaction is dominated by dispersion forces and that chemical bonding does not occur under ambient conditions.

Finally, the small differences among adsorption sites reflect the uniform, nonpolar nature of the graphene lattice. The optimized geometries confirm that the water molecule remains nearly intact after adsorption, with no evidence of surface structural distortion. In this context, the interaction is best described as physisorption, with negligible electronic rearrangement beyond weak polarization. These results also indicate that surface modifications—such as the introduction of structural defects, heteroatom dopants, or oxygen-containing functional groups—could substantially alter the adsorption landscape, increasing interaction strength and potentially enhancing the hydrophilicity of graphene-based materials. Such tunability is particularly relevant for applications in water purification, sensing, and catalysis.

## Conclusions

In this study, we investigated how a single water molecule interacts with pristine graphene using DFT calculations with the VDW-C09 functional. The results indicate that the interaction is weak, primarily governed by van der Waals forces, with adsorption energies ranging from −93 to −145 meV. Among all configurations analyzed, the most stable arrangement corresponds to the Down orientation of the water molecule positioned above the center of the hexagonal ring, with an adsorption energy of −145 meV and an equilibrium distance of approximately 3.27 Å. In this geometry, the water molecule is oriented toward the graphene surface, which favors slightly stronger dispersion interactions. The H-bond and Up configurations are less stable, presenting smaller binding energies and longer molecule–surface separations. The calculated equilibrium distances, between 2.6 and 3.3 Å, confirm that the molecule remains physically adsorbed, without forming chemical bonds or significantly altering the graphene surface. The small variation in adsorption energy across different sites also reflects the homogeneous and non-reactive nature of pristine graphene, consistent with its experimentally observed hydrophobic behavior. Overall, these results provide a clear and intuitive understanding of how water interacts with an ideal graphene sheet. Although the interaction is weak, it serves as a foundation for exploring how defects, dopants, or functional groups could be introduced to tailor graphene’s surface chemistry. Such modifications can enhance water adsorption, opening new opportunities for applications in water purification, catalysis, and nanoscale fluid control.

## Data Availability

No datasets were generated or analyzed during the current study.
